# Simulation and experimental study of local high frequency resistance distribution in proton exchange membrane fuel cells under steady and dynamic conditions

**DOI:** 10.1016/j.heliyon.2024.e40648

**Published:** 2024-11-22

**Authors:** Xin Zhan, Jiaqi Sun, Feng Ding, Xiaozhi Xu, Wei Song, Zhigang Shao

**Affiliations:** a Fuel Cell System and Engineering Laboratory, Key Laboratory of Fuel Cells & Hybrid Power Sources, Dalian Institute of Chemical Physics, Chinese Academy of Sciences, Dalian, Liaoning, 116023, China; b University of Chinese Academy of Sciences, Beijing, 100039, China; c University of Science and Technology of China, Hefei, Anhui, 230026, China

**Keywords:** Proton exchange membrane fuel cell, Local high frequency resistance distribution, Mechanical degradation, Stress analysis, Three-dimensional multiphase model

## Abstract

Proton-exchange membrane (PEM) dry–wet variation during PEM fuel cell (PEMFC) operation markedly affects PEMFC lifespan. Therefore, deeper insights into the mechanical degradation mechanism of PEM require analysis of the membrane dry–wet change process. The stress changes caused by PEM dry–wet variations may induce mechanical failure. In practice, although high-frequency resistance (HFR) is often used to indirectly characterize PEM dry–wet degrees, numerical simulation can effectively analyze the mass transfer inside a PEMFC for parameters that are difficult to measure directly experimentally (such as membrane water content). Additionally, three-dimensional (3D) model validation requires more comprehensive experimental methods. In this study, we validate the simulated resistance distribution results through local electrochemical impedance spectroscopy. We also discuss the mass- and heat-transfer distribution characteristics under different current densities and analyze the influence of these characteristics on local HFR distribution variations. The calculated HFR distribution results show a certain degree of agreement with the experimental results. Due to the membrane self-humidification effect, the position of the minimum HFR value shifts upstream of the cathode at a high current density. The observed change in membrane stress results from variations in membrane water and temperature distributions with changes in current density. Steady-state HFR distribution analysis to uncover the membrane dry–wet variations caused by load changes—which would affect the HFR distribution changes—reveal that the HFR at the air outlet and the membrane dry–wet variations are more pronounced than at other locations due to the influence of reaction rate and gas velocity. Overall, this study demonstrates that 3D simulations can reliably predict the local HFR distribution of PEMFCs, enabling membrane stress variation analysis and providing guidance for fuel cell design and operation.

## Highlights

• Local high-frequency resistance (HFR) distribution of PEMFCs is predicted.
• Local HFR distribution tests can be used to analyze membrane stress distribution.
• Studied proton-exchange membrane fuel cells (PEMFCs) in steady and dynamic conditions.
• Temperature distribution variation considerably effects mechanical degradation.
• Degradation at the air outlet can be severe during load changes.

## Graphical abstract

**Figure:**
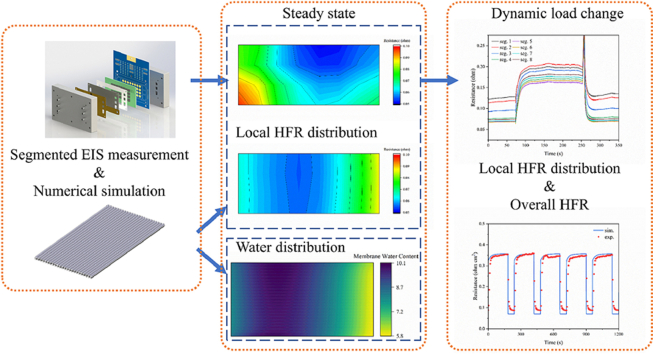

## 1 Introduction

In terms of global warming and increasingly serious environmental pollution, fuel cells are considered an ideal solution to energy shortages and environmental pollution, which are attributed to their high efficiency and low emissions [[1], [2], [3]]. Proton exchange membrane (PEM) fuel cells (PEMFCs) have been broadly applied in many fields, such as transportation, mobile equipment, small power stations, and the military, owing to their low working temperature, fast response, good stability, and the use of pure hydrogen as fuel without causing environmental pollution [[4], [5], [6]]. However, cost and lifetime are key factors limiting widespread commercial application [7,8].

A fuel cell always experiences dynamic load variations during operation under actual vehicle operating conditions [9]. However, frequent load change is a key factor affecting fuel cell lifespan among all vehicle operating conditions. Due to the membrane water absorption–desorption during the working cycle of PEMFCs, the constrained membrane frequently expands and contracts with water content variations, thus generating mechanical stress and membrane degradation. Membrane degradation manifests in several aspects, such as creep, fatigue, and the formation of pinholes, cracks, and tears, ultimately leading to performance degradation and serious failure [10,11]. Therefore, to prevent PEM failure from endangering PEMFCs' safe operation, a deep understanding and analysis of the membrane's mechanical degradation are necessary to alleviate fuel cell degradation [[12], [13], [14], [15]].

The study of water management in fuel cells involves mass transfer and water transport inside the cells. Visualization techniques such as neutron imaging [16] and X-ray imaging [17] have been employed to study water transport characteristics inside fuel cells in the past few years. Although visualization technology can be utilized to directly monitor water distribution in fuel cells, its equipment is expensive, technically demanding, and difficult to perform quantitative analysis. Moreover, neutron imaging cannot characterize dynamic changes and therefore cannot evaluate dynamic water-content changes [18]. Electrochemical impedance spectroscopy (EIS) reflects the internal working state and electrochemical behavior of PEMFCs, among which high-frequency resistance (HFR) is the main experimental method for characterizing the membrane water content in situ [19,20]. For example, Toyota measured fuel cell humidity using AC impedance measurement [21], wherein the experimental results revealed negative correlations between impedance and water content in the fuel cells. Hao et al. [22] studied the effects of liquid water generation on fuel cell performance by measuring the HFR of a PEMFC. Gerteisen et al. [23] examined local current and HFR distributions using the segmented cell method. However, HFR was also unable to reflect the quantitative characterization of the water distribution in the involved PEMFC. Therefore, we can study the water transport characteristics inside the PEMFC through simulation to understand membrane stress changes intensively. Simulations can compensate for the deficiency of experimental research and save experimental time and cost, which markedly influences PEMFC design and optimization.

With the development of computer technology, numerical simulation has an indispensable status in the in-depth study of fuel cell issues. Notably, experimental validation remains an essential part of model research because it can reflect simulation result reliability. Among them, the common validation experimental method is the polarization curve [24]. However, for three-dimensional (3D) models, a comprehensive approach is required to validate the local parameter distribution in PEMFCs [[25], [26], [27]]. The parameter distribution of the PEMFC can be effectively measured by segmented measurement technology. The principle involves dividing the electrode into several segments on the activated surface and measuring the physical parameters of each segment independently, such as current and temperature [[28], [29], [30]]. Zhang et al. [31] studied the difference in current density distribution by changing loads and operating conditions and found good coherence between model predictions and experimental measurement results. Hao et al. [32] comprehensively verified the fuel cell multiphase model by combining current density and temperature distribution tests.

Transient research on fuel cells is also crucial as the conditions of automotive fuel cells are constantly changing during operation. Hasan et al. [33] studied membrane surface stress variations caused by dry–wet cycles under different working conditions and revealed that the strain caused by humidity changes in the membrane exceeded that caused by temperature changes. Kosakian et al. [34] studied dynamic changes in liquid water by establishing a 2D model. Based on this, the dynamic property and stability of the fuel cells were also investigated by polarization curve and voltage step experiments. Futter et al. [35] build a transient model for analyzing PEMFC performance and studied the PEMFC physical processes based on EIS simulation. However, because of the high computational difficulty of 3D models, most works only focus on the overall changes in fuel cells and lack discussion on local dynamic changes.

In summary, HFR can be employed to characterize membrane dry–wet changes and analyze its mechanical degradation mechanism. Although numerical simulation can be used to study the internal mass-transfer characteristics of PEMFC, experimental validation markedly contributes to 3D numerical simulation research. However, only a few PEMFC modeling studies have validated models with sufficient data and in-depth analysis, especially from the perspective of local HFR distribution. In this study, we studied the differences in HFR distribution under different current densities by establishing a 3D model of PEMFC using COMSOL Multiphysics and validating it through local HFR tests. In addition, based on the dry–wet changes in PEM under the dynamic load cycle of the PEMFC, we studied the changes in HFR distribution during the dynamic change process. For the first time, we discussed the intrinsic relation between local HFR distribution and mechanical failure, providing a deeper understanding of the effect of membrane wet–dry variations on local mechanical degradation and offering guidance for membrane failure diagnosis.

The innovations of this paper are as follows: (1) We used the local EIS test method as a verification method for the 3D model for the first time and discussed the intrinsic relationship between the local HFR distribution and mechanical failure. (2) By combining numerical simulation and local EIS test technology to characterize and analyze the stress distribution of the membrane, it will solve the problem of difficult characterization of internal stress. (3) The results of the dynamic HFR study found that the HFR and water content changed significantly at the cathode outlet, which theoretically explained why mechanical degradation severe at this location.

## 2 Theoretical model and simulation method

### 2.1 Model assumptions

First, we establish a 3D multiphase fuel cell model with the following assumptions:

1. First, due to the low temperature and low back pressure of PEMFCs under operation, the gas in the system can be regarded as an ideal gas and mixed evenly.
2. Hydrogen and oxygen are assumed not to pass through the PEM; that is, no gas crossover occurs.
3. As cathode catalyst layer (CCL) construction markedly affects the overall reaction, CCL is regarded as a spherical agglomeration model.
4. The liquid water produced by the reaction is evenly coated outside the agglomerate, which consequently forms a water film.
5. As the simulated operating conditions have low humidity, we can assume that the water in the gas flow channels is in the gaseous phase, and no phase change occurs.

### 2.2 Governing equations

The model used in this study contains some governing equations, including mass conservation, momentum conservation, species conservation, membrane water, liquid water, proton conservation, and electron conservation. The nomenclature and abbreviations used in this paper are listed in the supplementary material. Table 1 lists the PEMFC characteristics and operating condition parameters. The governing equations are as follows:

Mass conservation equation: The mass conservation for gas mixtures is calculated using the following equation (solved in the flow channel, GDLs, and CLs):

(1) $$\frac{\partial }{\partial t}\left(\varepsilon \left(1-s\right)\rho_{g}\right)+\nabla \cdot \left(\rho_{g}{\vec{u}}_{g}\right)=S_{m}\text{.}$$

Momentum conservation equation: The Navier–Stokes equations are used to describe momentum conservation in the flow channel, GDLs, and CLs.

(2) $$\frac{\partial }{\partial t}\left(\frac{\rho_{g}{\vec{u}}_{g}}{\varepsilon \left(1-s_{lq}\right)}\right)+\nabla \cdot \left(\frac{\rho_{g}{\vec{u}}_{g}{\vec{u}}_{g}}{\varepsilon^{2}{\left(1-s_{lq}\right)}^{2}}\right)=-\nabla p_{g}+\mu_{g}\nabla \left(\nabla \left(\frac{{\vec{u}}_{g}}{\varepsilon \left(1-s_{lq}\right)}\right)+\left(\frac{{\vec{u}}_{g}^{T}}{\varepsilon \left(1-s_{lq}\right)}\right)\right)-\frac{2}{3}\mu_{g}\nabla \left(\nabla \left(\frac{{\vec{u}}_{g}}{\varepsilon \left(1-s_{lq}\right)}\right)\right)+S_{u}\text{.}$$

Species conservation equation: The transfer process of gas molecules from the flow channel to the GDLs and CLs is calculated via the Maxwell–Stefan equation; where *i* represents H_2_, O_2_, and H_2_O.

(3) $$\frac{\partial }{\partial t}\left(\varepsilon \left(1-s_{lq}\right)\rho_{g}Y_{i}\right)+\nabla \cdot \left(\rho_{g}{\vec{u}}_{g}Y_{i}\right)=\nabla \cdot \left(\rho_{g}D_{i}^{eff}\nabla Y_{i}\right)+S_{i}\text{.}$$

Liquid phase volume fraction conservation equation: Equation (4) is the governing equation used to calculate the liquid water saturation of GDLs and CLs following Darcy's law.

(4) $$\frac{\partial }{\partial t}\left(\rho_{lq}\varepsilon s_{lq}\right)+\nabla \cdot \left(\iota \rho_{lq}{\vec{u}}_{g}\right)=\nabla \cdot \left(\rho_{lq}D_{lq}\nabla s_{lq}\right)+S_{lq}\text{.}$$

The driving force of liquid water in porous structures is described by capillary pressure. In this study, the porous media is considered hydrophobic; that is, the capillary pressure is <0. The diffusion coefficient of liquid water in porous media is affected by capillary pressure, which follows the Leverett function.

(5) $$D_{lq}=-\frac{K_{lq}}{\mu_{lq}}\frac{dp_{c}}{ds_{lq}}\text{.}$$

(6) $$p_{c}=p_{g}-p_{lq}=\sigma \;\cos\;\theta {\left(\frac{\varepsilon }{K}\right)}^{0.5}J\left(s_{lq}\right)\text{.}$$

(7) $$J\left(s_{lq}\right)=\left\{ \begin{array}{l} 1.42\left(1-s_{lq}\right)-2.12{\left(1-s_{lq}\right)}^{2}+1.26{\left(1-s_{lq}\right)}^{3}\;\theta <90^{\circ }\\ 1.42s_{lq}-2.12{s_{lq}}^{2}+1.26{s_{lq}}^{3}\;\theta >90^{\circ } \end{array}\right.\text{.}$$

where *θ* is the contact angle, which is dependent on the material interface hydrophilicity. *σ* represents the surface tension between the liquid and gas, which is calculated as follows:

(8) σ=−0.0001676T+0.1218.

In PEM and CL, the membrane water content conservation equation: As the pressure on both sides of the cathode and anode is equal and constant, the membrane water considers only the electroosmotic resistance and the back diffusion of water.

(9) $$\frac{\rho_{mem}}{EW}\frac{\partial }{\partial t}\left(\varepsilon_{ion}\lambda \right)+\nabla \cdot \left(\frac{n_{d}}{F}i_{m}\right)=\frac{\rho_{mem}}{EW}\nabla \cdot \left(D_{mw}^{eff}\nabla \lambda \right)+S_{mw}\text{,}$$

where $D_{mw}$ denotes the diffusion coefficient of H_2_O, which dissolved in the membrane, and $n_{d}$ is the electroosmotic resistance coefficient. They are both functions of the membrane water content, which are calculated as follows:

(10) $$D_{mw}=\left\{ \begin{array}{l} 3.1\times 10^{-7}\lambda \left(\exp\left(0.28\lambda \right)-1\right)\exp\left(\frac{-2346}{T}\right)\;0<\lambda <3\\ 4.17\times 10^{-8}\lambda \left(161\;\exp\left(-\lambda \right)+1\right)\exp\left(\frac{-2346}{T}\right)\;3\leq \lambda <17\\ 4.1\times 10^{-10}{\left(\frac{\lambda }{25}\right)}^{0.15}\left(1+\tanh\left(\frac{\lambda -2.5}{1.4}\right)\right)\;\lambda \geq 17 \end{array}\right.\text{.}$$

(11) $$n_{d}=\frac{2.5\lambda }{22}\text{.}$$

The heat energy conservation applies to all regions of the PEMFC:

(12) $$\frac{\partial }{\partial t}\left({\left(\rho C_{p}\right)}_{s,fl}^{eff}T\right)+\nabla \cdot \left({\left(\rho C_{p}\right)}_{fl}^{eff}{\vec{u}}_{g}T\right)=\nabla \cdot \left(k^{eff}\nabla T\right)+S_{T}\text{.}$$

Electron conservation is solved on GDLs and CLs, whereas proton conservation is solved on CLs and Mem.

(13) $$-\nabla \cdot \left(\kappa_{ele}^{eff}\nabla \phi_{ele}\right)=S_{ele}\text{.}$$

(14) $$-\nabla \cdot \left(\kappa_{ion}^{eff}\nabla \phi_{ion}\right)=S_{ion}\text{.}$$

Among them, the effective conductivity is corrected using the Bruggeman equation.

(15) $$\kappa_{\text{ele}}^{eff}=\kappa_{ele}\varepsilon^{1.5}\text{.}$$

(16) $$\kappa_{ion}^{eff}=\kappa_{ion}\varepsilon^{1.5}\text{.}$$

Proton conductivity is affected by membrane water content and temperature and is calculated using the following function:

(17) $$\kappa_{ion}=\left(0.5139\lambda -0.326\right)\exp\left(1268\left(\frac{1}{303.15}-\frac{1}{T}\right)\right)\text{.}$$

Table 2 lists the source terms of the above governing equations (see Table 1). Butler–Volmer equation shows the relation between current density and overpotential, which is calculated using Equation (18).

(18) $$i_{a}=A_{cl,a}\left(1-s_{lq}\right)i_{0,a}{\left(\frac{\left(1-s_{lq}\right)\varepsilon_{ACL}c_{H_{2}}}{c_{H_{2}}}\right)}^{0.5}\left(\exp\left(\frac{2\alpha_{a}F}{RT}\eta_{act}^{a}\right)-\exp\left(-\frac{2\left(1-\alpha_{a}\right)F}{RT}\eta_{act}^{a}\right)\right)\text{.}$$

**Table 1:** Characteristic parameters and operating conditions.

Parameter	Value
Channel width; depth; rib width	1.0; 1.0; 1.0 mm
Thicknesses of membrane; CL; GDL	25; 10; 200 μm
Volume fraction of GDLs	0.6
Ionomer to carbon weight ratio	0.7
Intrinsic permeabilities of CL; GDL	*K*^*0*^ = 6.2 × 10^−13^; 6.2 × 10^−12^ m^2^
Density of CL; GDL; membrane	*ρ* = 1000; 1000; 1980 kg m^−3^
Thermal conductivity of membrane; CL; GDL	*k* = 0.95; 1.0; 1.0 W m^−1^ K^−1^
Electrical conductivity of CL; GDL	*κ* = 200; 1250 S m-1
Stoichiometry ratio	*ξ*_*a*_ = 1.5; *ξ*_*c*_ = 2.5
Relative humidity of inlet gas	*RH* = 30 %
Inlet gas temperature; operating temperature	*T*_*0*_ = 353.15 K
Operating pressure	*p* = 0.5 bar

**Table 2:** Source terms.

Source term	Unit
$S_{m}=\left\{ \begin{array}{l} -S_{v-l}+S_{v-m}-\frac{i_{a}}{2F}M_{H_{2}}\;\text{Anode}\;\text{CL}\\ -S_{v-l}+S_{v-m}-\frac{i_{c}}{4F}M_{O_{2}}\;\text{Cathode}\;\text{CL}\\ -S_{v-l}\;\text{GDLs},\text{Channels} \end{array}\right.$	kg m^−3^ s^−1^
$S_{u}=-\frac{\mu_{g}}{Kk_{g}}{\vec{u}}_{g}\;\text{CLs},\text{GDLs}$	kg m^−2^ s^−2^
$S_{H_{2}}=-\frac{i_{a}}{2F}M_{H_{2}}\;\text{Anode}\;\text{CL}$, $S_{O_{2}}=-\frac{i_{c}}{4F}M_{O_{2}}\;\text{Cathode}\;\text{CL}$	kg m^−3^ s^−1^
$S_{H_{2}O}=\left\{ \begin{array}{l} -S_{v-l}+S_{v-m}\;\text{CLs}\\ -S_{v-l}\;\text{GDLs},\text{Channels} \end{array}\right.$	kg m^−3^ s^−1^
$S_{lq}=\left\{ \begin{array}{l} S_{v-l}\;\text{GDLs},\text{Channels}\\ \frac{i_{c}}{2F}M_{H_{2}O}+S_{v-l}\;\text{CLs} \end{array}\right.$	kg m^−3^ s^−1^
$S_{v-m}=\gamma_{v-m}\frac{\rho_{mem}}{EW}\left(\lambda -\lambda_{eq}\right)\left(1-s_{lq}\right)\;\text{CL}$	mol m^−3^ s^−1^
$S_{ele}=\left\{ \begin{array}{l} -i_{a}\;\text{Anode}\;\text{CL}\\ i_{c}\;\text{Cathode}\;\text{CL} \end{array}\right.$, $S_{ion}=\left\{ \begin{array}{l} i_{a}\;\text{Anode}\;\text{CL}\\ -i_{c}\;\text{Cathode}\;\text{CL} \end{array}\right.$	A m^−3^
$S_{v-l}=\left\{ \begin{array}{l} \gamma_{v-l}\varepsilon \left(1-s_{lq}\right)\left(c_{H_{2}O}-c_{sat}\right)M_{H_{2}O}\;c_{H_{2}O}>c_{sat}\\ \gamma_{l-v}\varepsilon s_{lq}\left(c_{H_{2}O}-c_{sat}\right)M_{H_{2}O}\;c_{H_{2}O}<c_{sat} \end{array}\right.$	kg m^−3^ s^−1^

Considering the complex structure of CL, a spherical agglomeration model was adopted for the cathode [36].

(19) $$i_{c}=A_{cl,c}4F\frac{p_{O_{2}}}{H_{O_{2}}}{\left(\frac{1}{E_{agg}k_{agg}}+\frac{r_{agg}+\delta_{agg}+\delta_{w}}{\gamma r_{agg}}\right)}^{-1}\text{.}$$

where $p_{O_{2}}$ represents the oxygen partial pressure and $H_{O_{2}}$ represents the Henry coefficient. γ denotes the diffusion rate of oxygen in the ionomer film and the water film, which cover the ionomer surface:

(20) $$\gamma =\frac{\gamma_{m}\gamma_{w}}{\gamma_{m}+\gamma_{w}},\gamma_{m}=\frac{a_{m}D_{O_{2},m}}{\delta_{agg}},\gamma_{w}=\frac{a_{w}D_{O_{2},w}}{\delta_{w}}\text{.}$$

*E*_*agg*_ is the effective factor of the spherical catalyst and *M*_*T*_ is the dimensionless parameter Thiele's modulus;

(21) $$E_{agg}=\frac{1}{M_{T}}\left(\frac{1}{\tanh\left(3M_{T}\right)}-\frac{1}{3M_{T}}\right)\text{.}$$

(22) $$M_{T}=\frac{r_{agg}}{3}\sqrt{\frac{k_{agg}}{D_{O_{2},agg}^{eff}}}\text{.}$$

where *k*_*agg*_ is the reaction rate coefficient, calculated by equation (23).

(23) $$k_{agg}=\frac{a_{agg}}{4F}\frac{i_{0,c}^{ref}}{c_{O_{2}}^{ref}}\left(\exp\left(\frac{-\alpha_{c}F}{RT}\eta_{c}\right)-\exp\left(\frac{\left(1-\alpha_{c}\right)F}{RT}\eta_{c}\right)\right)\text{.}$$

The specific surface area of the ionomer *a*_*m*_ and the specific surface area of the ionomer covered by the water film *a*_*w*_ are as follows:

(24) $$a_{m}=\frac{m_{Pt}A_{s}}{t_{cl}}\frac{\left(1-\varepsilon_{cl}\right)}{\varepsilon_{Pt/C}}\text{.}$$

(25) $$a_{w}=a_{m}{\left(1+\frac{\delta_{w}}{r_{agg}}\right)}^{2}\text{.}$$

The specific surface area per unit mass of platinum *A*_*s*_, the ionomer film thickness *δ*_*agg*_ and the water film thickness *δ*_*w*_ are as follows:

(26) $$A_{s}=\left(227.79f^{3}-158.57f^{2}-201.53f+159.5\right)\times 10^{3}\text{.}$$

is calculated using the following formula:

(27) $$\delta_{agg}=r_{agg}\left({\left(\frac{1-\varepsilon_{cl}}{\varepsilon_{Pt/C}}\right)}^{1/3}-1\right)\text{.}$$

(28) $$\delta_{w}=\sqrt[{3}]{{\left(r_{agg}+\delta_{agg}\right)}^{3}+\frac{3s\varepsilon_{cl}}{4\pi N_{agg}}}-\left(r_{agg}+\delta_{agg}\right)$$

The number of spherical particles per unit volume *N*_*agg*_ is calculated as follows:

(29) $$N_{agg}=\frac{1-\varepsilon_{cl}}{\frac{4}{3}\pi {\left(r_{agg}+\delta_{agg}\right)}^{3}}\text{.}$$

The overpotential is defined as follows:

(30) $$\eta_{i}=\varphi_{ele}-\varphi_{ion}-E_{i}^{eq}\text{.}$$

The output voltage *E*_*cell*_ of the PEMFC is calculated using the following formula:

(31) $$E_{cell}=E_{0}-\eta_{a}+\eta_{c}-i_{M}R_{M}\text{,}$$

where *E*_0_ represents the open circuit voltage:

(32) $$E_{0}=E_{c}^{eq}-E_{a}^{eq}\text{.}$$

Herein, ohmic resistance is used to represent HFR. Ohmic resistance includes proton resistance and electron resistance and is calculated based on Joule heat as follows:

(33) $$R_{ele}=\frac{1}{i^{2}A}\int_{V}\sigma_{ele}^{eff}\nabla \phi_{ele}\cdot \nabla \phi_{ele}dV\text{.}$$

(34) $$R_{ion}=\frac{1}{i^{2}A}\int_{V}\sigma_{ion}^{eff}\nabla \phi_{ion}\cdot \nabla \phi_{ion}dV\text{.}$$

The stress change due to humidity and temperature changes is calculated as follows:

(35) $$\varepsilon_{ij}^{S}=\alpha^{RH}\left(RH-RH_{0}\right)\delta_{ij}\text{.}$$

(36) $$\varepsilon_{ij}^{T}=\alpha^{T}\left(T-T_{0}\right)\delta_{ij}\text{.}$$

Table 3 lists the electrochemical parameters and transmission coefficients.

**Table 3:** Parameter of electrochemical kinetics and transport parameters.

Parameter	Correlation/value	Unit
Transfer coefficient	$\alpha_{a}=\alpha_{c}=1$	
Reference exchange current density	$i_{0,a}=1000;\;i_{0,c}=1$	A cm^−2^
Reference hydrogen, oxygen concentrations	$c_{H_{2}}^{ref}=56.4;\;c_{O_{2}}^{ref}=3.39$	mol m^−3^
Activation energy in oxygen reduction reaction	$E_{c}=67000.0$	J mol^−1^
Liquid water dynamic viscosity	$\mu_{lq}=2.414\times 10^{-5}\times 10^{247.8/\left(T-140.0\right)}$	m^2^ s^−1^
Gas mixture dynamic viscosity	$\mu_{g}=\sum_{i}\frac{x_{i}\mu_{i}}{\sum_{j}x_{j}\varphi_{ij}}$	m^2^ s^−1^
Evaporation, condensation rates	$\gamma_{v-l}=\gamma_{l-v}=100$	s^−1^
Membrane water and water vapor phase change rate	$\gamma_{v-m}=1.0$	s^−1^
Henry's constant of oxygen at the ionomer surface	$H_{O_{2}}=0.1\times \exp\left(14.1-666/T\right)$	Pa mol^−1^ m^−3^
Entropy change of reaction in anode	$\Delta S_{a}=130.68$	J mol^−1^ K^−1^
Entropy change of reaction in cathode	$\Delta S_{c}=32.55$	J mol^−1^ K^−1^

### 2.3 Boundary conditions

Set the boundary conditions of temperature, pressure, volume flow rate, and reactant concentration at the gas inlet of the cathode flow channel.

(37) $$\begin{array}{l} T=T_{0},p_{c}=1\;\text{bar},{\text{sccm}}_{c}=\frac{\xi_{c}IA}{4F}\times \frac{RT}{0.21p_{c}}\text{,}\\ x_{H_{2}O,c}^{0}=\frac{p_{sat}RH_{c}}{p_{c}},x_{O_{2}}^{0}=0.21\left(1-x_{H_{2}O,c}^{0}\right),x_{N_{2}}^{0}=0.79\left(1-x_{H_{2}O,c}^{0}\right) \end{array}\text{.}$$

The boundary conditions at the anode gas inlet are the same as those at the cathode.

(38) $$\begin{array}{l} T=T_{0},p_{a}=1\;\text{bar},{\text{sccm}}_{a}=\frac{\xi_{a}IA}{2F}\times \frac{RT}{p_{a}}\text{,}\\ x_{H_{2}O,2}^{0}=\frac{p_{sat}RH_{a}}{p_{a}},x_{H_{2}}^{0}=1-x_{H_{2}O,c}^{0} \end{array}\text{.}$$

Dirichlet boundary conditions are set at the flow channel/GDL and CL/MEM interfaces. The heat-transfer rate at the bipolar plate boundary is calculated as follows:

(39) $$\dot{Q}=hA_{wall}\left(T_{0}-T\right)\text{.}$$

The PEMFC model was solved using the commercial software COMSOL Multiphysics 6.0. The MEA dimensions of the computational domain have 10 units in the *x*-direction and 200 units in the *y*-direction. The channel uses a triangular network with a maximum unit size of 0.3 mm. The *z*-direction has 50 units, and the computational domain is meshed using a sweeping method. A total of 151,680 grids were included. The computer configuration includes a processor (Intel (R) Core (TM) i5-10505 CPU @ 3.20 GHz) and 64 GB DDR RAM.

## 3 Experimental section

The anodic Pt loading of the catalyst-coated membrane (CCM) was 0.2 mg cm^−2^, and the cathodic Pt loading was 0.4 mg cm^−2^. A Nafion 211 PEM was used as the electrolyte. The CCM was sandwiched between two GDLs, which were fixed on both sides with a polyester frame. The MEA preparation was then completed by hot pressing at 140 °C and 0.1 MPa for 90 s. The MEA size was 100 mm × 50 mm (effective reaction area of the cell was 50 cm^2^).

**Fig. 1:**
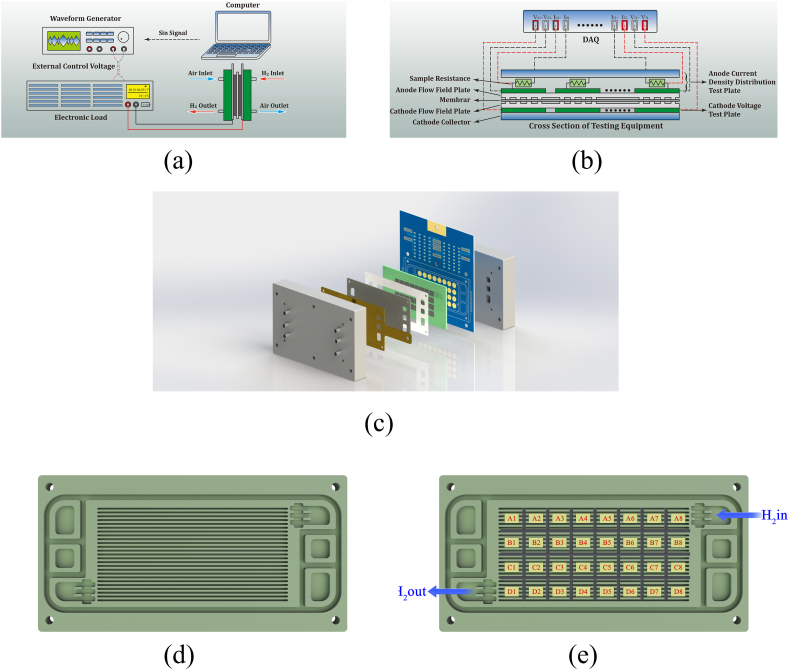
Experimental process and equipment diagram: (a) EIS test equipment and process, (b) PCB equipment diagram for local EIS test, (c) assembly diagram of fuel cell and PCB, (d) parallel flow field structure diagram, and (e) flow field segmentation method and numbering.

Graphite plates were employed as the flow field of both cathode and anode, with parallel flow fields on both sides (Fig. 1(c)), where the channel width and depth were 0.1 cm and the rib width was 0.1 cm. As shown in Fig. 1(d), the printed circuit board (PCB) was divided into 32 segments, where the channel direction was divided into eight sections, and the vertical channel direction was divided into four sections. Fig. 1(a) and (b) show the primary equipment employed to conduct local HFR evaluation tests: waveform generator (Rigol Technologies, DG2072), an electronic load (ITECH, IT8811, and Kikusui PLZ664W), and a data acquisition card (DAQ; Vkinging, VK7016).

Pure hydrogen was used as the fuel gas and fresh air as the oxidizing gas in all experiments, with a bypass humidifier ensuring humidity. The PEMFC was always maintained at a working temperature of 80 °C and an operational back pressure of 50 kPa. The cathode and anode inlet humidity was 30 %. The anodic and cathodic stoichiometric ratios were 1.5 and 2.5, respectively, at 800 mA cm^−2^ and 6 and 10, respectively, at 200 mA cm^−2^. The gas input direction was counterflow.

During the local EIS measurement, some voltage signal data were inputted from the computer to the waveform generator. The voltage signals control the electronic load and induce rapid changes in the PEMFC load current. The voltage signal is calculated using the following formula to form a sine signal:

(40) V=ksin(2πft+b),

where *k* is a constant determined by considering the voltage range of the load external programming control and the range of the electronic-load constant-current mode. *F* represents the frequency at which the EIS measurement is expected to be conducted. *B* represents the corresponding phase angle. The signal is transmitted to the external voltage control of the electronic load after the waveform generator receives it. Consequently, the voltage is output to the electronic load. The electronic load applies an excitation current to the PEMFC guided by voltage control. Herein, the frequency was set to 1000 Hz and the excitation amplitude was 5 %. Once the DAQ starts working, the anode current-density-distribution test board collects current and voltage distribution signals from various areas. The current signal is determined by calculating the voltage drop caused by the sampling resistor, which is generated by the disturbed current calculated using the following equation:

(41) $$I=\frac{V}{R_{sample}}\text{,}$$

where *I* denotes the current passing through the sampling resistor, *V* indicates the voltage drop across the sampling resistor, and *R*_sample_ denotes the resistance value of the sampling resistor. After the anode current-density-distribution test board captures the negative electrode voltage of the PEMFC, the voltage is combined with the positive electrode voltage captured by the cathode voltage test board to obtain differential voltage signals in various regions.

Once the excitation current and voltage data in different regions are collected by the DAQ, the data are transmitted to the computer. Subsequently, the data in the time domain are transformed to those in the frequency domain through Fourier transform, thus obtaining the voltage and current values at the corresponding frequencies. Finally, the local EIS data for each region can be obtained using the following equation:

(42) $$Z=\frac{V\left(f\right)}{I\left(f\right)}\text{.}$$

The numbering scheme for each segment is shown in Fig. 1(d).

## 4 Results and discussion

The results of the numerical simulations and experiment are presented and discussed in the following sections. We first performed experiments based on the input parameters of the simulations and obtained local HFR distribution data to validate the 3D multiphase model. On this basis, we further studied the changes in HFR distribution under dynamic dry–wet changes and analyzed them from the changes in membrane water-content distribution.

### 4.1 Validation of HFR distribution

Conducting comprehensive and convincing validation requires comparing the spatial distribution of characteristic parameters. To validate the credibility of the simulation results, we compared the predicted ohmic resistance (*R*_ohm_) distribution with the experimental data from real test results. To control a single variable, the inlet gas stoichiometric ratios were 1.5/2.5 and 6.0/8.0 at the anode/cathode for 800 and 200 mA cm^−2^, respectively. Due to the excitation current of the waveform generator, the real current densities were 220 and 830 mA cm^−2^. The high-frequency impedance distribution in the experiment was measured through 8 × 4 regions. Therefore, for the convenience of comparison, we coarsen the calculated results into 8 × 4 data points by averaging the impedance values of each region.

**Fig. 2:**
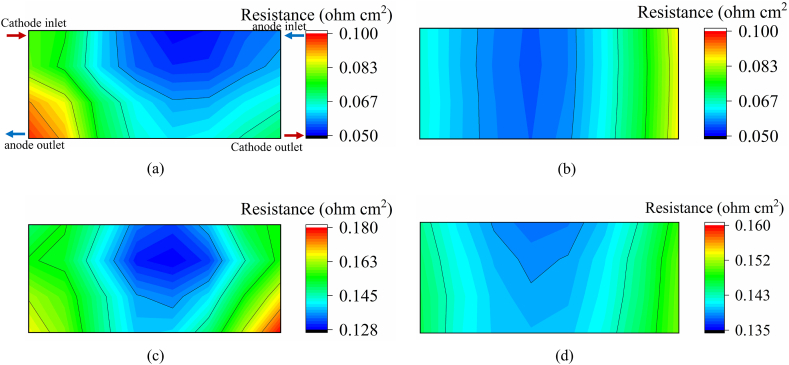
Experimental and simulated local HFR distributions at different current densities: (a) experimental result at 800 mA cm^−2^, (b) simulated result at 800 mA cm^−2^, (c) experimental result at 200 mA cm^−2^, and (d) simulated result at 200 mA cm^−2^

As shown in Fig. 2, the simulated local HFR distribution results agree with the experimental results to some degree. The local HFR distribution shows a similar trend under two different current loads. In particular, the low HFR region is at the location of the middle region of the bipolar plate. Fig. 2(b) and (d) show the ohmic resistance (*R*_ohm_) distributions under different simulated current densities. The region of the low local *R*_ohm_ is located in the middle region and downstream of the cathode under a low current load. However, at high current loads, the low local resistance region is transferred upstream to the cathode. The observed deviations between the simulated and experimental results may have stemmed from variations in fluid distribution between the real flow field and the simulation, but the deviation is accepted. Additionally, since the contact resistance between electrodes is neglected during the simulation, the simulated impedance value is always smaller than the actual measured value.

### 4.2 Effect of current density

Regarding the differences in HFR distribution under different current densities, we analyze the reasons in terms of membrane water content distribution and oxygen concentration distribution.

**Fig. 3:**
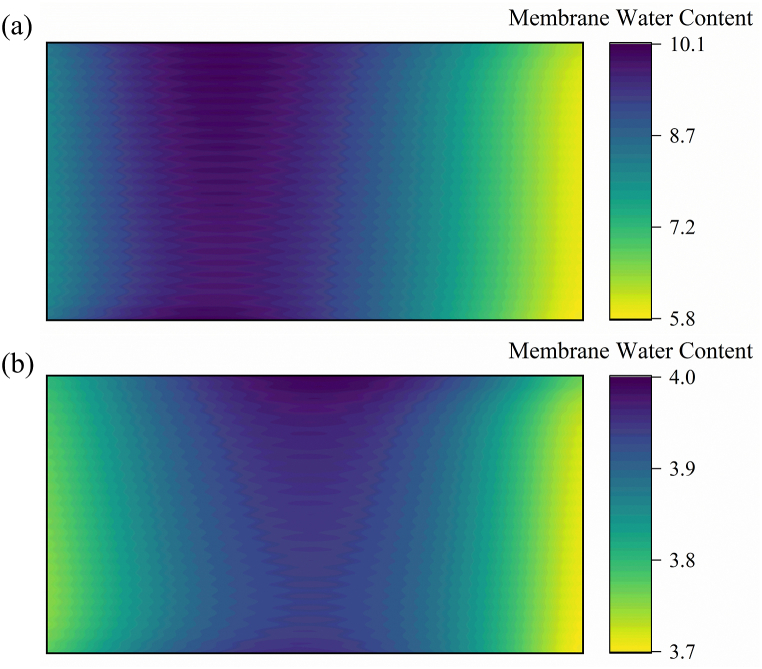
Membrane water-content distribution at the Mem/CCL interface at two different current densities: (a) 800 and (b) 200 mA cm^−2^

Fig. 3 shows the distribution of membrane water content (λ) when the PEMFC operates at 200 and 800 mA cm^−2^. The results indicate that the distribution of membrane water content and R_ohm_ distribution present an opposite trend; that is, the location with a large impedance has a small λ. When the fuel cell is operated at 200 mA cm^−2^, the upstream area near the two gas inlets has a lower water content and higher *R*_ohm_ than the middle region of the cell due to the high gas flow rate and low humidification. Under PEMFC operation at 800 mA cm^−2^, whereas the self-humidification effect of the electrochemical reaction induces increased water content [37], oxygen consumption in the middle and downstream regions triggers an elevated partial pressure of H_2_O in the gaseous phase [38]. Moreover, due to the fixed flow rate, the stoichiometric ratio when the fuel cell operates at 800 mA cm^−2^ is smaller than that at 200 mA cm^−2^, and the water removal rate is low. Therefore, the local *R*_ohm_ of the middle and upstream regions of the cathode of the fuel cell is small under a high current load.

**Fig. 4:**
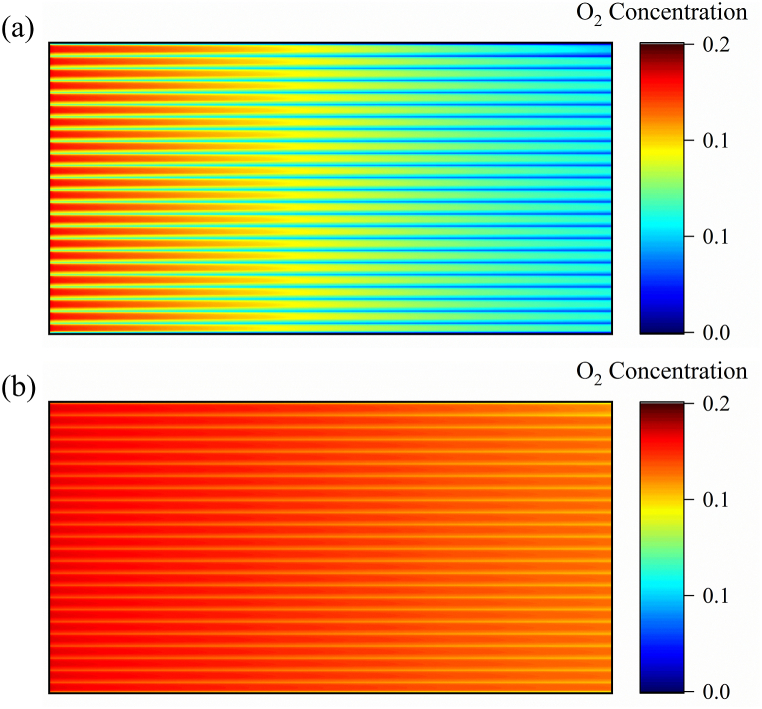
Oxygen concentration distribution at the Mem/CCL interface at two current densities: (a) 800 and (b) 200 mA cm^−2^

Fig. 4 shows the oxygen concentration distribution at different current densities, illustrating a decrease in the oxygen concentration downstream of the cathode as the current density increases. The minimum oxygen mole fraction at a high current density (800 mA cm^−2^) is only 0.02, whereas it is 0.11 at a low current density (200 mA cm^−2^). Since the same input velocity is used at different current densities, the stoichiometric ratio is small at a high current density. Consequently, the oxygen supply downstream of the cathode is insufficient at high loads.

Significantly, the high overlap between impedance and water distribution indicates that the in-plane stress distribution of the fuel cell can be monitored by local EIS testing technology. Humidity changes directly affect membrane stress changes, resulting in membrane creep and fatigue, and the initiation and propagation of pinholes, cracks, and tears, which lead to performance degradation and catastrophic failure [39]. Equation (35) expresses the relation between the in-plane stress and humidity change [13,40], where *RH* and *RH*_0_ represent the local humidity and reference humidity, respectively (the average water content in the surface is selected as the standard); α_RH_ is the linear expansion coefficient; and *δ*_*ij*_ is the Kronecker increment.

In summary, the uneven distribution of water is the main cause of the unevenly distributed in-plane stress. Large differences in local water content can easily cause catalyst layer and membrane degradation [41]. Therefore, improving full cell performance and lifespan requires the development of PEMs with low humidity durability. Additionally, optimizing the uniformity of water distribution by changing the flow field structure can improve the membrane's mechanical durability.

**Fig. 5:**
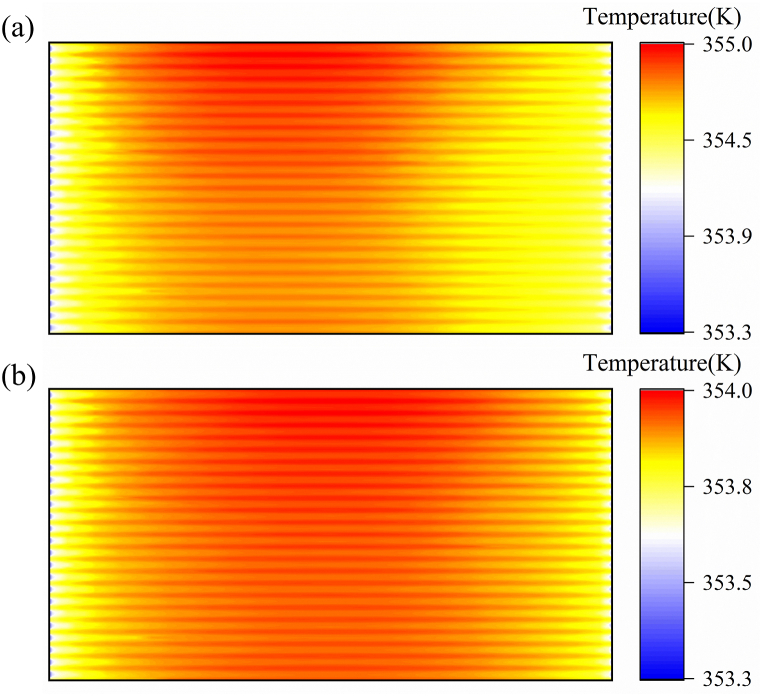
Temperature distribution at the Mem/CCL interface at two different current densities: (a) 800 and (b) 200 mA cm^−2^

Fig. 5 shows the temperature distribution under different current densities. The temperature increases with increasing current density, and the distribution becomes more uneven. This is because the electrochemical reaction generates reaction heat. From the above discussion, we proved that the temperature distribution at a high current density has a large temperature gradient due to the uneven mass distribution. Although increased temperature induces accelerated membrane degradation [42], it also affects the thermal and water management of PEMFCs [43]. Equation (36) shows the relation between temperature and stress changes. Although the effect of temperature difference on stress change is smaller than that of humidity, temperature remains a factor that cannot be ignored when considering its effect on mechanical degradation [11]. In summary, the difference in temperature distribution caused by load changes will cause uneven local stress distribution, which will affect the membrane's mechanical degradation. Therefore, the effect of temperature on mechanical stress is also one of the key points in future research on PEMFC durability. Moreover, temperature distribution can be utilized to guide the design of cooling channels and flow fields.

### 4.3 Study of the local impedance variation during dry–wet cycling

As fuel cells must continuously change loads in actual operation, this study also explores impedance changes during load changes. To effectively simulate the actual operating conditions of fuel cells, we used dynamic working conditions to simulate the membrane dry–wet changes during actual operation and then analyzed the mechanical damage to the membrane. In this study, we simulated the membrane's mechanical degradation behavior caused by the dry–wet variations by cycling high and low current densities [44]. The change in the overall HFR was obtained by varying the current density between 0.08 and 0.8 A cm^−2^.To save computational cost, we first simulated the dynamic changes in the *R*_ohm_ of the PEMFC by a simplified 2D model.

**Fig. 6:**
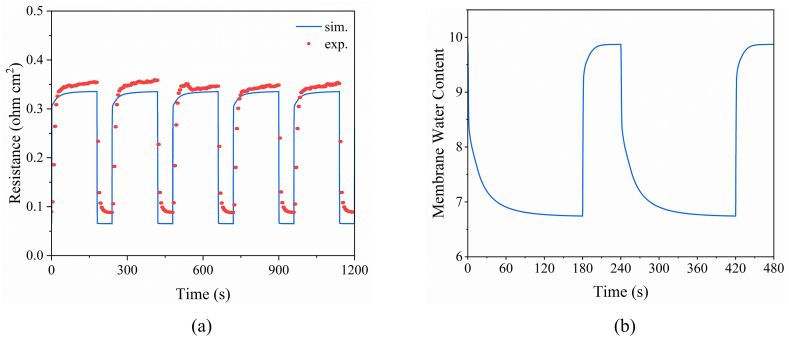
(a) Simulation results of dynamic HFR changes and their experimental validation. (b) Dynamic membrane water-content changes.

Fig. 6(a) shows the simulation results of the dynamic HFR changes and the experimental validation. The calculated dynamic variations in *R*_ohm_ are basically consistent with experimental results that revealed HFR changes of 0.08–0.35 Ω cm^2^ for a current density variation of 0.08–0.8 A cm^−2^. The membrane water-content variations affected the HFR changes (Fig. 6(b)). At high current density, electrolyte resistance decreases due to the self-humidification effect during the reaction process, which hydrates the membrane. However, since water exists in different states in the MEA [45], the membrane has slower dehydration than hydration.

Although the overall HFR change reflects the water content of the fuel cell, local degradation often causes the entire cell's degradation [14,46]. Particularly, the stress change caused by the variation in local water content markedly contributes to the local degradation of fuel cells. Therefore, we also studied the dynamic changes in HFR distribution. To simplify the difficulty of the experiment, we selected eight areas as test objects for the research. Seg. 1–8 correspond to A1–A8 in Fig. 1(b), respectively, where Seg. 1 represents the air inlet and Seg. 8 represents the hydrogen inlet. Since the minimum excitation current density is 20 mA cm^−2^, to reduce the influence of the excitation current, the minimum current density is set to 200 mA cm^−2^. A overshoot in tested HFR occurs at the moment of transition from low load to high load (t = 260 s) because the step rate of the current is much greater than 1000 Hz, substantially affecting the disturbance signal at the step point.

**Fig. 7:**
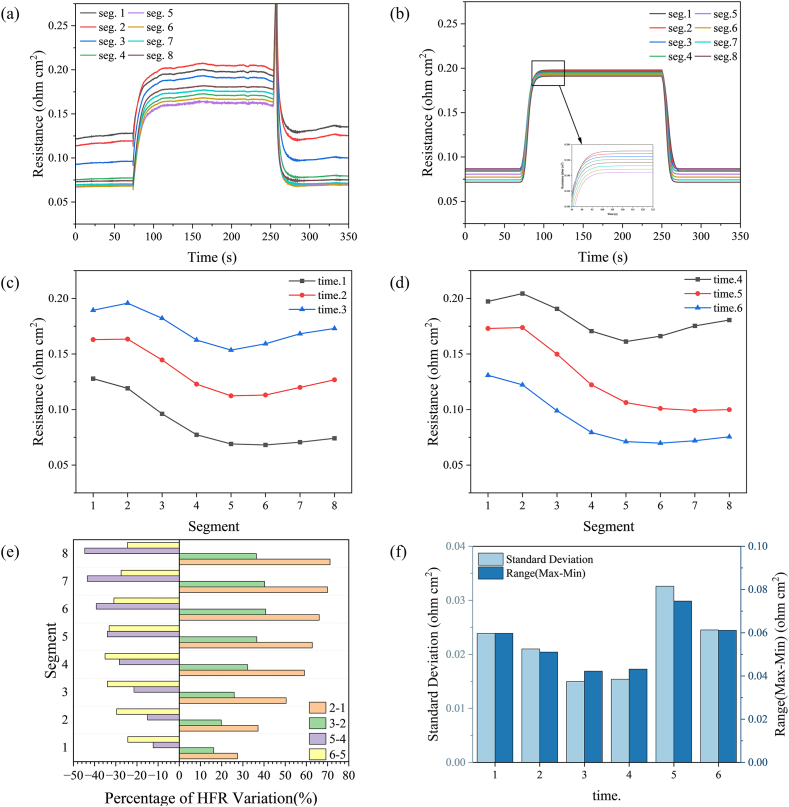
Changes in local HFR distribution: (a) experiment and (b) simulation; changes in HFR distribution at selected moments: (c) current density reduction process, (d) current density increase process, (e) HFR change degree, and (f) HFR standard deviation and range at selected moments.

In order to observe the HFR distribution characteristics at a selected time, HFR at different times in the experimental results was also selected for analysis (Fig. 7(c) and (d)). Times 1 to 6 correspond to 60, 80, 100, 240, 260, and 280 s in Fig. 7(a), respectively. Time.1 to time.3 represents the current density changes from high to low, while time.4 to time.6 is the opposite.

From Time.1 to Time.3, the local HFR distribution shows an increasing trend. The HFR minimum is always located in the middle of the flow field (Seg. 5), while the maximum gradually shifts to the right from the cathode inlet (from Seg. 1 to Seg. 2). In addition, the HFR variation has minimum and maximum values at the air inlet (Seg. 1) and the hydrogen inlet (Seg. 8), which are 0.07 and 0.11 Ω cm^2^, respectively. From Time 4 to Time 6, the local HFR distribution shows a decreasing trend. The minimum value of the HFR transfers from the middle of the bipolar plate (Seg. 5) to the cathode outlet (Seg. 8).

We calculated the HFR change rate at different times using the following formula [47]: $k_{m-n}=\frac{R_{i}-R_{0}}{R_{0}}\times 100\%$, where *m* − *n* represents the change rate from Time *n* to Time *m*. As shown in Fig. 7(e), during current density reduction, the change rate of the HFR at the cathode outlet (Seg. 8) is the largest and decreases along the cathode outlet to the inlet. The HFR change rate tends to decrease over time. However, a reversal of the HFR change rate occurred upon increasing the current density.

To analyze the uniformity of local HFR distribution at different times, we also calculated the range and standard deviation. The standard deviation is calculated using the following formula: $\sigma =\sqrt{\frac{1}{N}\sum_{i=1}^{N}{\left(R_{ij}-\bar{R}\right)}^{2}}$. As shown in Fig. 7(d), the standard deviation and range of the HFR distribution show a decreasing trend with decreasing current density. However, the standard deviation and range of Time 5 exceed those of Time 4 and Time 6 as the current increases. The reason is that Time 5 is at the moment of the HFR overshoot, and the change at this moment is peculiar.

**Fig. 8:**
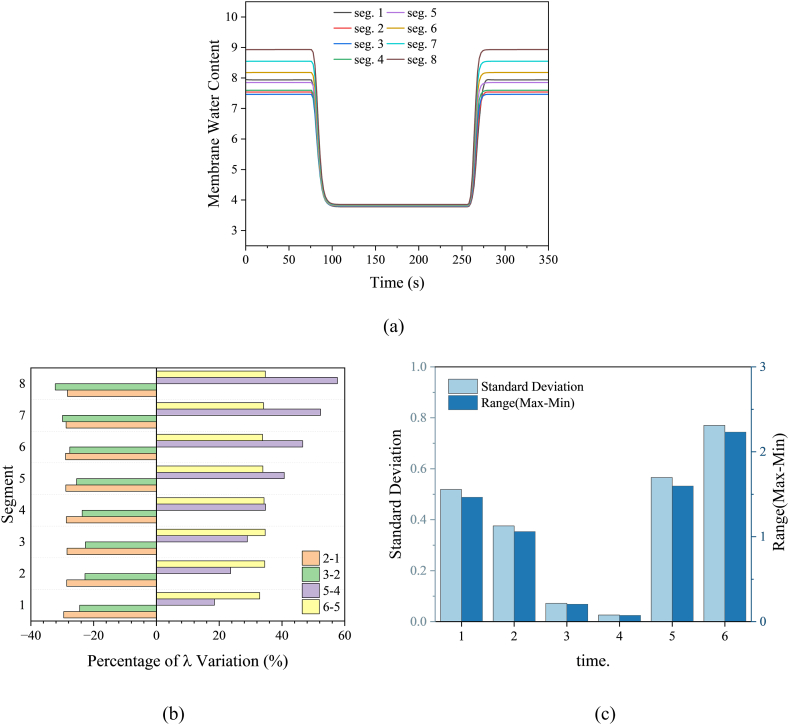
(a) Membrane water-content distribution during dynamic change. (b) Membrane water-content change rate. (c) HFR standard deviation and range at selected time.

The uneven distribution of local HFR is caused by the uneven distribution of membrane water content. As shown in Fig. 8, the membrane water-content distribution at a high current density is more uneven than that at a low current density, and the membrane water-content change rate at the cathode outlet (Seg. 8) exceeds that at other locations. Although the self-humidification effect is small at low current density and the local membrane water content is mainly affected by the dry input humidity under low humidification intake conditions, an increase in current density leads to more water production from the reaction, particularly accumulating at the cathode outlet. At a low current density, the water accumulated at the cathode end is considerably reduced until the water content across the entire flow channel stabilizes, with a uniform range of approximately 0.01. In summary, the HFR and water content at the cathode outlet of the PEMFC change the most, resulting in the largest stress change. Therefore, future research should focus more on optimizing the flow field structure to alleviate degradation at the cathode outlet.

## 5 Conclusion

We first combine 3D multiphase simulation and local EIS testing technology to study the high-frequency resistance distribution of PEMFC at different current densities and analyzed the mass- and heat-transfer characteristics of PEMFC. Based on the study of steady-state HFR, we also investigate HFR and water distribution characteristics during fuel cell load variation. The main conclusions are as follows:

(1) The simulated *R*_ohm_ distribution results agree well with the experimentally tested HFR distribution data. The HFR distribution shows an opposite trend to the water distribution in the membrane, that is, the location with low resistance is located at the location with a high water content.
(2) Owing to the increase in water generated by the electrochemical reaction and the use of a constant flow rate, the stoichiometric ratio at 800 mA cm^−2^ is smaller than at 200 mA cm^−2^. Therefore, at a high current density, the local *R*_ohm_ value near the mid-upstream region in the cathode is small.
(3) Although the effect of temperature change on mechanical degradation is much smaller than that of water content change, it cannot be ignored. Therefore, studying the impact of temperature distribution on mechanical stress changes is also a key point in future research on PEMFC durability.
(4) During the load cycle, the HFR value and membrane water content undergo greater changes and higher change rates at the air outlet than at other locations, leading to greater stress variation in this area. Therefore, optimizing the flow field design to mitigate membrane degradation at the cathode outlet should be prioritized.

## CRediT authorship contribution statement

**Xin Zhan:** Writing – original draft, Methodology, Conceptualization. **Jiaqi Sun:** Validation, Methodology. **Feng Ding:** Methodology, Investigation. **Xiaozhi Xu:** Methodology, Investigation. **Wei Song:** Supervision, Project administration. **Zhigang Shao:** Funding acquisition.

## Data availability

Data will be made available on request.

## Declaration of competing interest

The authors declare that they have no known competing financial interests or personal relationships that could have appeared to influence the work reported in this paper.
