# Supplementary material for: Simulation and experimental study of local high frequency resistance distribution in proton exchange membrane fuel cells under steady and dynamic conditions
Source: Heliyon. 2024 Nov 22;10(23):e40648. doi: 10.1016/j.heliyon.2024.e40648 (PMC11647849; doi:10.1016/j.heliyon.2024.e40648)
Supplement: Multimedia component 1 [file mmc1.docx]

**Nomenclature**

$A$ cell geometric area (m^2^)

$c$ molar concentration (mol m^-3^)

$C_{p}$ specific heat (J kg^-1^ K^-1^)

$D$ mass diffusivity (m^2^ s^-1^)

$EW$ equivalent weight of membrane (1100 g mol^-1^)

$F$ Faraday’s constant (96,487 C mol^-1^)

$h$ latent heat (J kg^-1^)

$I$ current density (A cm^-2^)

$i$ reaction rate (A cm^-3^)

$i_{0}$ exchange current density (A m^-3^)

$k$ thermal conductivity (W m^-1^ K^-1^)

$K$ permeability (m^2^)

 mass flow rate (kg s^-1^)

$M$ molecular weight (g mol^-1^)

$n_{d}$ electro-osmotic drag coefficient (H_2_O per H^+^)

$p$ pressure (Pa)

 heat transfer rate (W)

$R$ universal gas constant (8.314 J mol^-1^ K^-1^)

$RH$ relative humidity

$s$ volume fraction

$S$ source terms

$t$ time (s)

$T$ temperature (K)

$\vec{u}$ velocity (m s^-1^)

$V$ electrical potential (V)

$x$ mole fraction

*Greek letters*

$\alpha$ transfer coefficient

$\gamma$ water phase change rate

$\varepsilon$ porosity

$\zeta$ water transfer rate (s^-1^)

$\eta$ overpotential

$\theta$ contact angle

$\iota$ interfacial drag coefficient

$\kappa$ electrical conductivity (S m^-1^)

$\lambda$ water content in membrane

$\mu$ dynamic viscosity (kg m^-1^ s^-1^)

$\xi$ stoichiometry ratio

$\rho$ density (kg m^-3^)

$\sigma$ surface tension (N m^-1^)

 electrical potential (V)

*Subscripts and superscripts*

$a$ anode

$c$ cathode

$cell$ cell characteristic

$CL$ catalyst layer

$eff$ effective

$ele$ electronic

$eq$ equilibrium

$mw$ membrane water

$g$ gas phase

$GDL$ gas diffusion layer

$H_{2}$ hydrogen

$ion$ ionic

$lq$ liquid water

$mem$ membrane

$O_{2}$ oxygen

$ref$ reference state

$vp$ water vapor


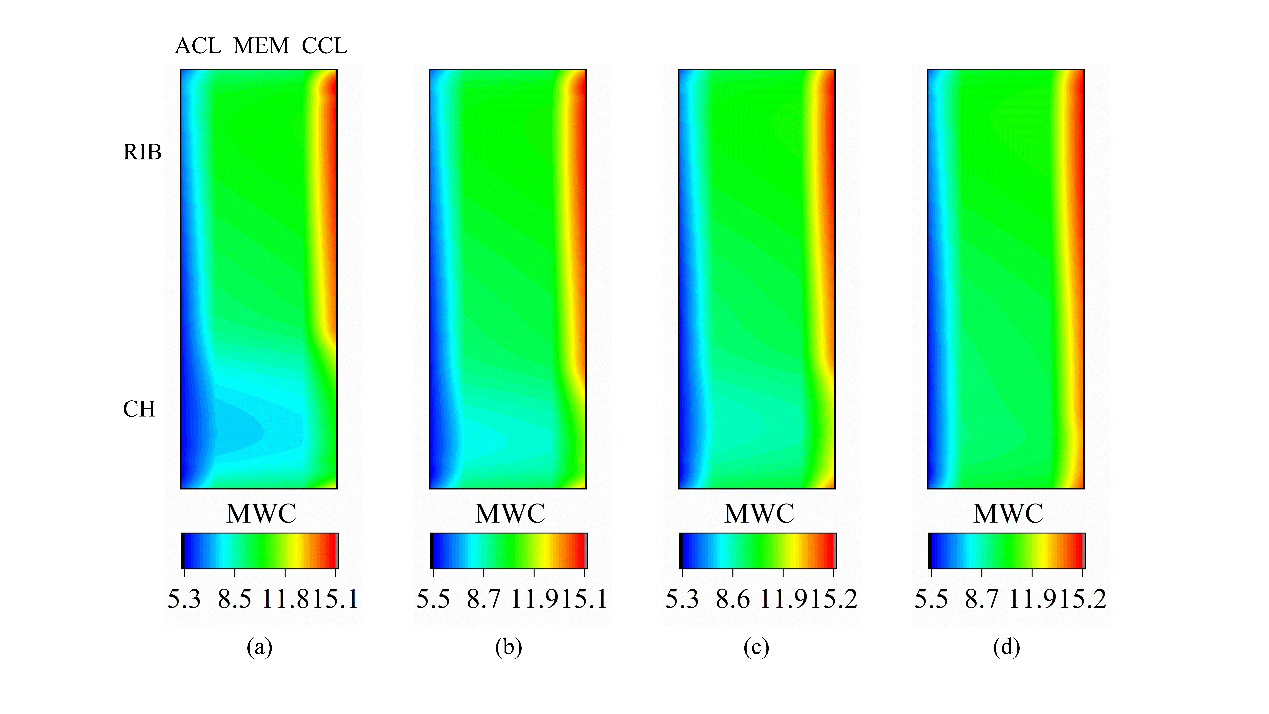


Figure 1 Schematic diagram of water distribution in the membrane electrode section at
0.8A cm^-2^: (a) 240 s inlet, (b) 240 s outlet, (c) 260 s inlet, and (d) 260 s outlet.


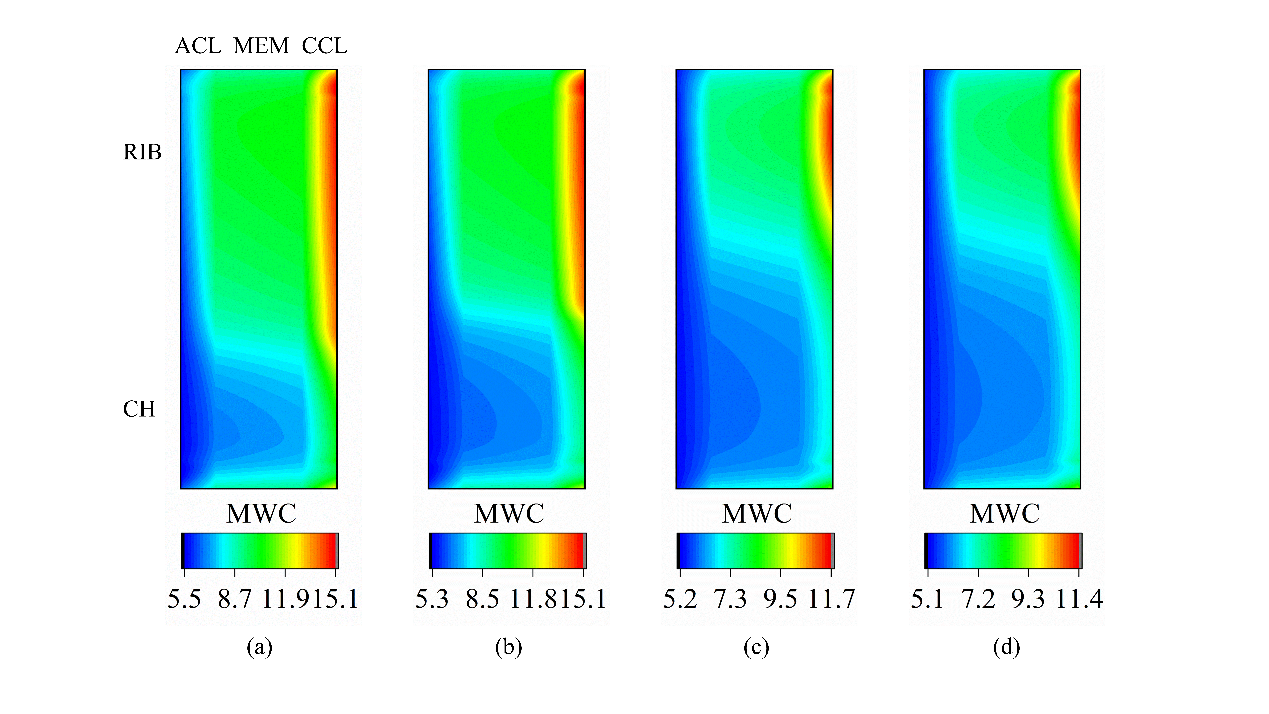


Figure 2 Schematic diagram of water distribution in the membrane electrode section at
0.08A cm-2: (a) 60 s inlet, (b) 60 s outlet, (c) 80 s inlet, and (d) 80 s outlet


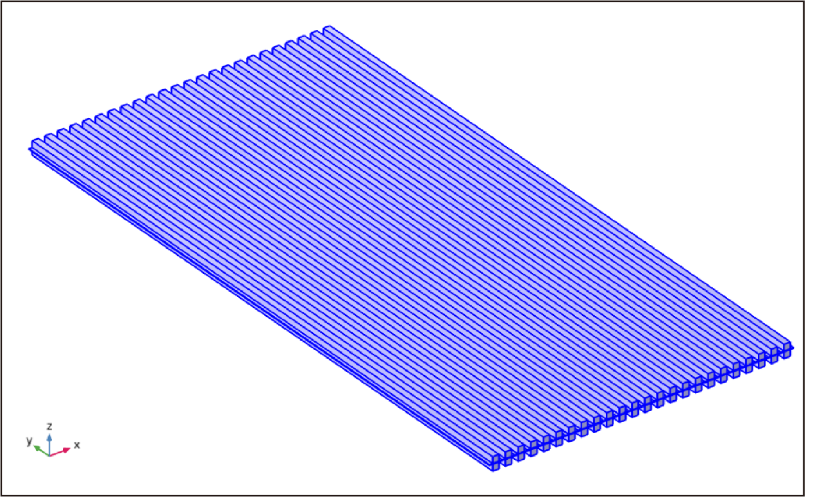


Figure 3 Fuel cell geometry model





Figure 4 Grid independence verification





Figure 5 Experimental validation of polarization curve
